# Adult airway management in ICU and regular floors: A prospective study as part of a healthcare improvement initiative in a tertiary care center

**DOI:** 10.1371/journal.pone.0341543

**Published:** 2026-01-27

**Authors:** Carine Zeeni, Mohamed Al Jazzar, Wissam Maroun, Thuraya HajAli, Marc Moukarzel, Rawad Jamaleddine, Patrick Maroun, Marie T. Aouad

**Affiliations:** 1 Department of Anesthesiology, American University of Beirut Medical Center, Beirut, Lebanon; 2 Department of Anesthesiology and Pain Medicine, Henry Ford Health, Detroit, Michigan, United States of America; 3 Quality, Accreditation, Risk Management Office, American University of Beirut Medical Center, Beirut, Lebanon; Imam Abdulrahman Bin Faisal University College of Medicine, SAUDI ARABIA

## Abstract

**Background:**

Despite recent advancements, airway management outside the operating room (OR) remains a challenging task and is associated with a high rate of complications. The first pass success rate at our institution was low. We developed a standardized multidisciplinary protocol to improve it as part of a healthcare improvement initiative.

**Methods:**

Following the implementation of the protocol, this prospective observational study was conducted on patients over 18 years old requiring intubation by anesthesia providers on regular floors and in intensive care units. The primary outcome was the first-attempt success rate of endotracheal intubation. Secondary outcomes included total number of attempts, time to successful intubation, equipment and medications used, and occurrence of adverse events such as severe hypotension (systolic blood pressure (SBP) ≤80 mmHg), severe hypoxemia (SpO2 ≤ 85%), and cardiac arrest.

**Results:**

All 199 patients were successfully intubated, with 184 patients (92.5%) intubated at first attempt. Twenty-four patients had a baseline SBP ≤ 80 mmHg, with 35 additional patients experiencing a drop in blood pressure during intubation. Thirty-eight patients had a baseline SpO_2_ ≤ 85%, with 11 additional patients experiencing desaturation during intubation. Two patients had cardiac arrest during intubation and were resuscitated. Multivariable logistic regression identified SpO2 ≤ 85% as the only predictor of hypoxemia (OR: 22.85, 95%CI: 9.07–57.55, P < 0.001) and baseline hypotension as the only predictor of hypotension during intubation (OR: 32.43, 95%CI: 7.14–147.33, P < 0.001).

**Conclusion:**

Implementing a standardized multidisciplinary protocol that includes highly experienced anesthesia providers and the use of muscle relaxants resulted in a very high first-attempt intubation success rate in critically ill patients, with the persistence of a high incidence of adverse events. Our successful approach may inform other policymakers about best practices for safe intubation in high-risk patients, highlighting the need for strategies to mitigate hemodynamic and respiratory complications and encouraging them to implement airway healthcare improvement initiatives in their institutions.

## Introduction

Managing airways outside the operating room (OR) is inherently complex and challenging. Non-OR intubations involve various factors that add complexity to the procedure beyond merely identifying difficult airways [1]. These span from logistics to patient-related factors, with a large proportion presenting for intubation with hemodynamic instability, respiratory compromise, or the presence of gastric contents [2], all of which contribute to higher complication rates particularly during repeated intubation attempts [3–6]. Improved first-attempt success rate is significantly associated with a reduction in major peri-intubation events [4]. Therefore, specific healthcare improvement initiatives should be undertaken to maximize first-attempt success rates [7–10]. Numerous guidelines for non-OR airway management exist to assist practitioners along with a recently published review on the subject [11,12]. The MACOCHA score helps assess airway difficulty in intensive care units (ICUs) [13]. However, airway management challenges extend beyond technical difficulty; a more holistic approach as represented by the Montpellier intubation protocol outlines the steps to improve ICU intubation outcomes [10]. Despite these resources, studies continue to indicate high complication rates in non-OR settings [3,14]. Variability in first-attempt success rates and adverse events throughout literature highlight the multifactorial nature of the issue, underscoring the inconsistency of resources and practices across healthcare institutions which makes it difficult to implement a standardized approach without considering one’s specific institutional capabilities [14–16].

The American University of Beirut Medical Center (AUBMC), a tertiary care facility in a lower-to-middle-income country, experienced significant variation in practices concerning non-OR airway management, like other institutions [14]. Between 2010 and 2015, data from our hospital revealed that the success rate of 1,932 non-OR intubations performed by non-anesthesiology providers using a non-standardized protocol was 70%. In response to a sentinel event and the recognition of a suboptimal intubation success rate, the Quality, Accreditation, and Risk Management (QARM) program collaborated with the Department of Anesthesiology and Pain Management to develop and implement a standardized care bundle for airway management in locations outside the operating room, excluding the Emergency Department (ED) following a Plan-Do-Study-Act cycle. As a first step, a task force commissioned by the QARM program identified gaps in the management of non-OR airways, summarized as follows: intubations were performed by non-anesthesiology providers with limited intubation experience, intubations were approached as isolated events, lacking a readily available qualified team to address complications, most intubations were performed with minimal sedation and without neuromuscular blocking drugs (NMBDs), the only available equipment was direct laryngoscopy, and the threshold to call for expert help was not clearly identified. A literature search confirmed that these challenges are common themes shared by many institutions globally [11,14,17]. Under the leadership of the anesthesiology department, a standardized multidisciplinary protocol incorporating the task force recommendations was created and adopted as a second step (S1 Table). Extensive training was provided to all stakeholders including presentations of the protocol followed by question-and-answer sessions and mock intubation drills.

As a third step, we designed a prospective observational study to evaluate the outcomes of endotracheal intubations for critically ill patients in ICUs and on regular floors and assess our practices after protocol implementation, aiming to compare our success and complication rates to those reported in the literature.

Our primary objective was to determine the first-attempt success rate of endotracheal intubation. Secondary objectives included assessing the incidence of adverse events and identifying their predictors.

We hypothesized that implementing a structured multidisciplinary airway management initiative outside the operating room would be associated with a high first-attempt intubation success rate.

## Materials and methods

This single-center prospective observational study was conducted at AUBMC between March 2, 2021, and September 26, 2023. Ethical approval was obtained from the Institutional Review Board (IRB) of the American University of Beirut on February 19, 2021 (Protocol ID: BIO-2020–0049). The study was registered on clinicaltrials.gov before patient enrollment (NCT04769154, principal investigator: Dr. Marie Aouad, date of registration: 23/02/2021, https://clinicaltrials.gov/study/NCT04769154). Written informed patient consents were waived by the IRB at the American University of Beirut as all data were recorded in a manner whereby the identity of subjects could not be ascertained directly or through identifiers linked to the subjects. This manuscript adheres to the SQUIRE 2.0 guidelines [18].

Adult patients aged more than 18 years who required intubation by an anesthesia provider, on regular floors and in ICUs were included between March 2021 and September 2023. All included patients were classified as American Society of Anesthesiology physical status IV. Since emergency medicine physicians have core privileges in endotracheal intubation, anesthesiologists were not involved with ED intubations; therefore, these were excluded from the study.

All airway code activations during the study period represented emergency airway encounters and a standardized institutional protocol was applied uniformly across all cases. All stakeholders involved received proper training and education to ensure adherence to the protocol. Non-OR airway management at AUBMC is regulated by the multidisciplinary protocol as follows: a code alert is activated for every intubation required outside the OR. The code team reports to the scene within five minutes (S2 Table).

A senior anesthesia resident (postgraduate year 4 (PGY4)) with documented experience of greater than 500 intubations performs the intubation procedure using direct laryngoscopy. A videolaryngoscope with hyper angulated blades is also immediately available. The operator may decide to start with videolaryngoscopy at their discretion. An attending anesthesiologist is immediately available on a consulting basis. Following preoxygenation with an O_2_ face mask, induction is initiated with propofol 1 mg/Kg IV or midazolam when baseline systolic blood pressure (SBP) is <100 mmHg (etomidate was unavailable during the study period). Rocuronium is routinely used for muscle relaxation, followed by rapid sequence intubation. Vasoactive drugs are prepared and readily available, with vasopressors given preemptively to patients with SBP less than 100 mmHg before intubation. Preoxygenation with 100% oxygen delivered via a tight-fitting face mask is used for all patients while induction medications and equipment are prepared. There is no predetermined duration of preoxygenation or SBP target due to the urgency of the intubations. For induction, midazolam is used if blood pressure is low, whereas propofol (1 mg/kg IV) is used otherwise, administered over approximately one minute. Vasopressors are also continued after intubation in the case of persistent hypotension. In the event of two failed intubation attempts, hospital protocol mandates the activation of the difficult airway code, which summons the difficult airway team consisting of an anesthesia attending and a senior otorhinolaryngology resident (S2 Table). Moreover, the difficult airway team will be activated proactively for patients identified or flagged as having a difficult airway (defined as previous difficult airway, or the presence of an airway pathology). Anesthesia providers involved in non-OR intubations follow a standardized bundle of steps that are summarized in S1 Table.

Data were prospectively collected by the code team anesthesia nurse during or immediately after the intubation ensuring reduction of recall bias. The data recorded in a de-identified manner on the structured data collection form included: patient characteristics, MACOCHA score, previous Cormack and Lehane score (used to define glottic view and interpreted as such: Grade 1 is a full view of the glottis, Grade 2 is a partial posterior view of the glottis, in Grade 3 only the epiglottis is visible, and Grade 4 is when no laryngeal structures are visible), location, number of months through PGY4, and baseline vital signs. The primary outcome was the first-attempt success rate, defined as percentage of successful intubation completed with a single insertion of the laryngoscope blade or the fiberoptic scope. Secondary outcomes included the total number of attempts, time to successful intubation, equipment and medications used, and the occurrence of adverse events such as severe hypotension (SBP ≤ 80 mmHg), severe hypoxemia (SpO2 ≤ 85%), and cardiac arrest. Failed intubation was defined as the inability to place the endotracheal tube after three attempts. The principal investigator conducted a twice weekly review of all airway forms and ensured the accuracy and completeness of the data by cross-referencing with the electronic medical record.

### Sample size calculation

The sample size was calculated using the estimation of a simple proportion method (https://epitools.ausvet.com.au/oneproportion). Studies have shown a first-attempt success rate of 75% [19]. Based on these results, a sample size of 100 patients was calculated, based on an estimated proportion of 70%, a desired precision estimate of 10%, a confidence level of 95%, and an estimated dropout rate of 20%. However, to improve the power of our study and allow for subgroup analysis and to explore associations, we elected to enroll 200 patients.

### Statistical analysis

All statistical analyses were conducted using IBM-SPSS (version 29, Armonk NY, USA).

Descriptive analysis was performed for patients’ demographics and clinical characteristics. Means and standard deviations were computed for continuous variables, and frequencies and percentages were calculated for categorical variables, along with 95% confidence intervals. Simple logistic regression was used to study the association between preoperative variables and hypotension and hypoxemia during intubation. Variables with a *P* value <0.25 were included in the multivariable logistic regression analysis. Multivariable logistic regression analysis was performed to identify the independent predictors of hypotension and hypoxemia during intubation while adjusting for other preoperative variables.

All models’ fits were assessed using the Hosmer and Lemeshow test. A *P* value <0.05 was considered statistically significant.

## Results

A total of 200 patients were recruited. One patient was excluded because of age of less than 18. Thus, 199 patients were included in this study. Baseline characteristics of the study participants are presented in Table 1.

**Table 1:** Baseline characteristics of the study participants.

	N = 199	95% CI
**Age (years)**	65.07 ± 16.67	[62.73, 67.40]
**Gender**		
**Male**	132 (66.3)	[59.7%, 72.9%]
**Female**	67 (33.7)	[27.1%, 40.2%]
**BMI (kg/m**^**2**^)	26.79 ± 6.27	[25.92, 27.67]
**BMI**		
**< 29 kg/m**^**2**^	146 (73.4)	[67.23, 79.51]
**≥ 30 kg/m**^**2**^	53 (26.6)	[20.49, 32.77]
**MACOCHA score (0–12)**	2.84 ± 2.66	[2.47, 3.21]
**Previous Cormack Lehane score**		
**I**	44 (22.1)	[16.3%, 27.9%]
**II**	15 (7.5)	[3.9%, 11.2%]
**III**	1 (0.5)	[0.0%, 2.8%]
**Unknown**	139 (69.8)	[63.5%, 76.2%]
**Location**		
**Floor**	43 (21.6)	[15.9%, 27.3%]
**ICU**	140 (70.3)	[64%, 76.7%]
**COVID ICU**	16 (8.0)	[4.3%, 11.8%]
**Postgraduate year 4 training**		
**< 4 months**	66 (33.2)	[26.6%, 39.7%]
**≥ 4 months**	133 (66.8)	[60.3%, 73.4%]
**Clinical presentation**		
**Cardiac arrest**	21 (10.6)	[6.3%, 14.8%]
**Respiratory failure**	138 (69.3)	[62.9%, 75.8%]
**Sepsis, deteriorating mental status**	40 (20.1)	[14.5%, 25.7%]

Data are presented as n (%) and mean ± standard deviation with 95% confidence intervals (CI).

BMI = body mass index, MACOCHA = Mallampati class, apnea, cervical spine limitation, mouth opening, coma, hypoxemia, non-anesthetist operator, ICU = Intensive Care Unit.

Respiratory failure was the leading cause of intubation in 138 patients (69.3%), while 40 patients (20.1%) required intubation for sepsis or deteriorating mental status, and the remaining 21 patients (10.6%) presented with cardiac arrest.

All patients were successfully intubated. One hundred eighty-four patients (92.5%) were successfully intubated at the first attempt. The difficult airway code was activated in 9 cases; 5 cases for anticipated difficult airway and 4 cases for more than two attempts at intubation. The first device used was direct laryngoscopy in 177 patients (88.9%), videolaryngoscopy in 18 patients (9.0%), and awake fiberoptic intubation in 4 patients (2.0%). Among the 18 cases where videolaryngoscopy was electively used as the first device, 7 cases were COVID-positive, 7 cases had a high MACOCHA score of more than 5, and in 4 cases it was the preference of the provider even in the absence of risk factors. Only one videolaryngoscopy case required more than a single attempt. All 4 fiberoptic intubation cases had laryngeal tumors/injuries; the difficult airway team was activated, and an attending anesthesiologist performed an awake fiberoptic intubation. One hundred seventy-one patients were successfully intubated with direct laryngoscopy, accounting for 85.9% of all cases. The mean time from code activation to successful intubation was 7.23 ± 5.62 minutes (Table 2).

**Table 2:** Outcome measures of the study participants.

	N = 199	95% CI
**Successful intubation**	199 (100.0)	[100,0%, 100.0%]
**Successful intubation from the first attempt**	184 (92.5)	[88.8%, 96.1%]
**Number of attempts**		
**1**	184 (92.5)	[88.8%, 96.1%]
**2**	11 (5.5)	[2.4%, 8.7%]
**3**	4 (2.0)	[0.6%, 5.1%]
**Anesthesia attending needed (difficult airway activated)**	9 (4.5)	[1.6%, 7.4%]
**First device used**		
**Direct laryngoscopy**	177 (88.9)	[84.6%, 93.3%]
**Video laryngoscopy**	18 (9.0)	[5.1%, 13.0%]
**Awake fiberoptic**	4 (2.0)	[0.6%, 5.1%]
**Intubation achieved with**		
**Direct laryngoscopy**	171 (85.9)	[81.1%, 90.8%]
**Video laryngoscopy**	24 (12.1)	[7.5%, 16.6%]
**Awake fiberoptic**	4 (2.0)	[0.6%, 5.1%]
**Time from code activation to successful intubation (min)**	7.23 ± 5.62	[6.43, 8.03]

Data are presented as n (%) and mean ± standard deviation with 95% confidence intervals (CI).

In 21 (10.6%) patients requiring airway management for cardiac arrest, advanced cardiac life support algorithms were followed. In 178 (89.4%) patients presenting with respiratory failure, deteriorating mental status or sepsis, hypnotics, NMBDs and vasoactive drugs were administered: rocuronium was used in 91% (95%CI [86.8%, 95.2%]) of these patients, midazolam in 58.4% (95%CI [51.2%, 65.7%]), propofol in 22.5% (95%CI [16.3%, 28.6%]), and either prophylactic or therapeutic vasopressors in 49.4% (95%CI [42.1%, 56.8%]). Of those who received vasopressors, 50% (95%CI [39.6%, 60.5%]) received phenylephrine, 27.3% (95%CI [18.0%, 36.6%]) received norepinephrine, 2.3% (95%CI [0.3%, 8.0%]) received epinephrine, and 20.4% (95%CI [12.0%, 28.9%)] received a combination of two or more vasopressors.

Twenty-four patients had a baseline SBP ≤ 80 mmHg. Thirty-five additional patients dropped their SBP to ≤80 mmHg during intubation. Thirty-eight patients had a baseline SPO_2_ ≤ 85%. Eleven additional patients dropped their SPO_2_ to ≤85% during intubation. Those patients with persistent hypotension were treated after intubation with vasopressor infusions as per institutional protocols. Two patients experienced cardiac arrest during intubation and were resuscitated (Table 3).

**Table 3:** Adverse events, excluding cardiac arrest patients.

	N = 178	95% CI
**Baseline SBP (mmHg)**	114.44 ± 29.85	[110.02, 118.85]
**Baseline DBP (mmHg)**	67.68 ± 18.99	[64.87, 70.49]
**Baseline MAP (mmHg)**	83.27 ± 21.74	[80.05, 86.48]
**Baseline SBP > 80 mmHg**	154 (86.5)	[81.5%, 91.5%]
**Baseline SBP ≤ 80 mmHg**	24 (13.5)	[8.5%, 18.5%]
**Baseline MAP > 65 mmHg**	141 (79.2)	[72.5%, 84.9%]
**Baseline MAP ≤ 65 mmHg**	37 (20.8)	[15.1%, 27.5%]
**SBP dropped ≤ 80 mmHg during intubation**	35 (19.7)	[16.1%, 29.4%]
**Baseline heart rate (beats/min)**	98.39 ± 23.83	[94.86, 101.91]
**Baseline SPO**_**2**_ **(%)**	90.64 ± 11.91	[88.88, 92.41]
**Baseline SPO_2_> 85%**	140 (78.7)	[72.6%, 84.7%]
**Baseline SPO_2_≤ 85%**	38 (21.3)	[15.3%, 27.4%]
**SPO**_**2**_ **dropped ≤ 85% during intubation**	11 (6.2)	[3.4%, 12.3%]
**Cardiac arrest during intubation**	2 (1.1)	[0.1%, 4.0%]

Data presented as n (%) and mean ± standard deviation.

DBP = Diastolic Blood Pressure, MAP = Mean Arterial Pressure, SBP = Systolic Blood Pressure.

There were no recorded deaths.

The multivariable logistic regression models were used to assess hypoxemia and hypotension, with variables showing *P* value <0.25 included in the models. The analysis revealed that hypoxemia at baseline (SPO2 ≤ 85%) was the only predictor of hypoxemia during intubation, with an odds ratio (OR) of 22.85 (95%CI: 9.07, 57.55), *P* < 0.001, after adjusting for the use of midazolam and residents’ level of experience (Table 4). Similarly, hypotension at baseline (SBP ≤ 80 mmHg) was the only predictor of hypotension during intubation, with an OR of 32.43 (95%CI: 7.14, 147.33), P < 0.001, after adjusting for the use of midazolam and hypoxemia at baseline (Table 4).

**Table 4:** Predictors of hypoxemia and hypotension during intubation.

Hypoxemia during intubation		
*Predictors*	OR^†^ [95%CI]	P-value
**Hypoxemia at baseline (SPO_2_≤ 85%)**		
No	Reference	
Yes	22.85 [9.07, 57.55]	<0.001
**Residents’ level of experience**		
Less experienced	Reference	
More experienced	1.21 [0.50, 2.88]	0.675
**Use of Midazolam**		
No	Reference	
Yes	0.79 [0.34, 1.82]	0.581
**Hypotension during intubation**		
***Predictors***	**OR**^**†**^ **[95%CI]**	**P-value**
**Hypotension at baseline (≤ 80 mmHg)**		
No	Reference	
Yes	32.43 [7.14, 147.33]	<0.001
**Hypoxemia at baseline (SPO_2_≤ 85%)**		
No	Reference	
Yes	1.36 [0.56, 3.31]	0.502
**Use of Midazolam**		
No	Reference	
Yes	1.02 [0.48, 2.13]	0.968

^†^ Adjusted odds ratio.

Both models had non-significant P values (>0.05) for Hosmer and Lemeshow test.

## Discussion

The main results of this prospective study are the very high success rate of endotracheal intubation at the first attempt recorded at 92.5% and the high incidence of hemodynamic instability that arises during this procedure, namely 33% of severe hypotension, and 27% of severe hypoxemia either at baseline or occurring during intubation, in addition to 1.1% incidence of cardiac arrest.

In the past decade, the literature has shown a pattern of improved airway management outside the OR. In a recent study by Nauka et. al, trends of success rate at the first attempt from studies on patients in ICUs or medical floors undergoing emergency endotracheal intubation over time were studied. Before 2015, intubation on first attempt ranged between 54% to 78%, and it later increased to a range of 75–83% [20]. Furthermore, it has been seen to be greater than 80% in the two most recent high-impact trials [21,22]. The global improvement in outcomes of airway management outside the OR can be attributed to several factors. Some are procedural in nature, such as the increased use of videolaryngoscopy, NMBD, and airway adjuncts [23–25]. Others are process-related, including the adoption of intubation bundles and the development of standardized protocols and procedures, most of which were part of healthcare improvement initiatives [6–10,26]. This highlights the importance of a comprehensive approach to improving outcomes which is consistent with our findings. Almost 15 years ago, Jaber et. al designed a comprehensive intubation protocol to improve success rates and minimize adverse events during intubation in the ICU [10]. The concept was later widely adopted and showed that its implementation resulted in a decreased incidence of adverse events [27]. Moreover, the publication of national and international airway management guidelines seems to be crucial in reducing the incidence of severe airway management complications [28].

Hall et. al reported a relatively low first-attempt intubation success rate of 73% [14]. Among the identified risk factors, the absence of NMBD use, and the limited intubating provider experience were associated with the highest odds ratios for requiring two or more intubation attempts (2.28 and 1.41 respectively). Of note, both factors were controlled in our study: NMBDs were used in 91%, and all intubations were performed by anesthesia-only providers with greater than 500 previous intubations. This might explain our very high first-attempt success rates compared to most of other major prospective studies looking at first-attempt success rate in adults outside the operating room (Table 5).

**Table 5:** Summary of recently published first-attempt intubation success rates in relation to operator experience and neuromuscular blocking drug use.

Author & year	First-attempt intubation success rate	Operator experience	Supplementary information	NMBD use
Zeeni et al. 2026	92.5%	>500 intubations (PGY 4 anesthesia)		>90%
Prekker et al. 2023 [29]	70.8%	Mean 50 intubations	<25 intubations: 53.9% success 25-100 intubations: 73.3% success >100 intubations: 83.5% success	96%
Hall et al. 2023 [14]	73.1%	55% inexperienced (PGY 1,2) 45% experienced (PGY 3, 4, 5, fellow or attending)	OR for ≥ 2 attempts: 2.28 if no NMBD 1.41 if inexperienced provider	63%
Garcia et al. 2023 [31]	78.8% 81.3% 83.6%	PGY1 PGY2 PGY3+	Only in ED	82.7%
Russotto et al. 2021 [4]	79.8%	26% ≤ 1 intubations/week 43% 2–5 intubations/week 19% 6–10 intubations/week 9% 11–20 intubations/week 3% > 20 intubations/week		75%
Driver et al. 2021 [21]	81.5%	Median of 60 intubations		>95%
Sakles et al. 2019 [26]	92.4%	13.7% PGY1 35% PGY2 51.2% PGY3	Only in ED	>99%
Janz et al. 2016 [30]	65.8%	<70 intubations	1 intubation: 47% 50 intubations: 62% 100 intubations: 77% 150 intubations: 89%	96%

NMBD = Neuromuscular Blocking Drug, PGY = Post Graduate Year, OR=odds ratio.

Intubations happening in ICUs and on the wards are typically performed by residents and fellows with a variety of backgrounds. Data from the most recent prospective studies published by the pragmatic critical care research group and other researchers have clearly demonstrated a strong association between intubating provider experience and first-attempt success rate. Prekker et. al’s study looking at intubations in ED and ICU showed an overall first-attempt success rate of 71% with a mean of 50 previous intubations performed, however, the absolute risk of successful intubation based on the number of previous intubations shot to 83.5% with greater than 100 previous intubations [29]. These results are corroborated by the work of Janz et. al that had an overall first-attempt success rate of 65.8% in the ICU, however, the first-attempt success rate based on the provider number of previous intubations extracted from their supplementary material shows that it can reach 89% when providers have greater than 150 intubations under their belts [30]. Garcia et. al showed that first-attempt success rates increased with the post-graduate level of intubating providers [31] and finally Russotto et. al showed that anesthesiologists had increased first-attempt success rates compared to others and being a staff physician compared to resident was independently associated with first-attempt intubation success [4] (Table 5). Our study was the only one where all providers were not only experienced but were exclusively anesthesiology practitioners. This factor, as supported by the finding of Russotto et. al [4], could have contributed to the high first-pass intubation success rates reported in our study.

Additionally, NMBD has emerged as a valuable intervention in improving the rate of successful intubation outside OR [32]. A direct relationship between NMBD use and first-attempt success rate of intubation is demonstrated in the literature; the use of NMBD occurred in 97% of the cases with 89% first-attempt success rate by Taboada et. al [3], in 75% of the cases with 80% first-attempt success rate by Russoto et. al [4], and in 62.5% of the cases with 73% first-attempt success rate by Hall et. al [14].

Our very high incidence of first-attempt success (92.5%) and the small number of failed cases (7.5%) did not provide enough statistical power to detect associations between potential risk factors and first-attempt success rate. Among factors previously shown to increase the first-attempt success rate is the routine use of videolaryngoscopy [23,29,33]. This is in contrast with other studies that did not show an improved outcome with videolaryngoscopy compared to direct laryngoscopy [30,34]. In our study, there was no association between the use of videolaryngoscopy and first-attempt success. The choice of the first device was left at the discretion of the provider, with an overwhelming majority choosing direct laryngoscopy (177 cases). Our results are in line with Janz’s data and his suggestion that the improvement in glottic view with videolaryngoscopy might matter the most for less experienced intubating providers [30] such as those in Prekker et. al’s study [29].

First-attempt intubation success rate is widely used as the primary outcome for assessing the quality of emergency endotracheal intubation outside the OR as it is specific, measurable, actionable and relevant. However, with the increased first-attempt intubation success rate in the past decade, the proportion of the outcome that can be modified through the implementation of novel interventions became minimal, leading to the need for much greater sample sizes to detect clinically significant differences between different patient cohorts and interventions. Thus, periprocedural complications recently emerged as valuable quality indicators in this context [20]. These indicators include hypotension, hypoxemia, aspiration of gastric contents, and cardiac arrest. The incidence of cardiac arrest in our study was1.1% compared to a range of 0.7 and 3.1% reported in other studies [4,21,32]. In our study, hypotension and hypoxemia were reported in a significant number of patients. Despite the preferential use of midazolam over propofol and the prophylactic use of vasopressors and preoxygenation, severe hypotension and hypoxemia were still prevalent. Thirty-five (19.7%) patients developed new episodes of hypotension compared to 24.6% reported by Russotto et. al [4]. Also, new episodes of severe hypoxemia occurred in 6.2% of the cases compared to an incidence ranging between 9.3 and 17.7% in the literature [3,4,32,35]. These studies excluded baseline hypotension and hypoxemia from their calculation undermining the magnitude of these side effects [3,4,32,35]. These percentages reflect the high incidence of adverse events occurring either at baseline or during intubation and underscore the critical nature of the patient population. In our study, baseline hypotension and hypoxemia were the only independent predictors of the occurrence of these complications during intubation.

There are several limitations to our study. First, it is a single-center study in an academic institution with a specific protocol and working environment which might limit the generalizability of our results. Second, the sample size and small number of unsuccessful intubations at the first attempt did not allow the exploration of associations between potential risk factors and the primary outcome. The limited number of outcome events constrained the regression models, and therefore, the results should be interpreted with caution. Third, the implementation of the bundle entailed numerous modifications; however, it was not possible to ascertain which specific component had the greatest impact on the improvement of our primary outcome. An additional limitation is that our institution has not yet adopted the recently standardized ATOM outcomes criteria [36]. Implementing these criteria in future practice would enable more consistent reporting and allow our results to be benchmarked against those of the wider scientific community. Moreover, due to the urgency of the intubations, there was no predetermined duration of preoxygenation, and induction and vasopressor administration were guided by clinical judgment rather than a formalized protocol, which may have contributed to variability in management.

## Conclusion

In conclusion, our study showed a very high first-attempt intubation success rate in critically ill patients, with the persistence of a high incidence of adverse events. We described the rigorous implementation of a standardized multidisciplinary protocol that includes highly experienced anesthesia providers and consistent NMBD use which resulted in a significantly improved first-attempt success rate. Our approach has the potential to inform policymakers regarding best practices for safe intubation of high-risk patients, emphasizing the importance of implementing strategies to mitigate hemodynamic and respiratory complications. As a next step, we intend to establish an airway registry and transition our healthcare initiative into a continuous quality improvement program, with the objective of reducing the incidence of adverse events associated with emergency intubation in outside the operating room setting.

## Supporting information

S1 Table Table 1 showing the American University of Beirut Medical Center intubation protocol. (PDF)

S2 Table Table 2 showing the code team members and difficult airway team members. (PDF)

S1 Checklist SQUIRE-2.0-checklist. (PDF)

## Abbreviations

AUBMC: American University of Beirut Medical Center
ED: Emergency Department
ICU: Intensive Care Unit
IRB: Institutional Review Board
NMBD: Neuromuscular blocking drugs
OR: Operating Room
PGY4: Postgraduate Year 4
SBP: Systolic blood pressure.
