# Supplementary material for: Adult airway management in ICU and regular floors: A prospective study as part of a healthcare improvement initiative in a tertiary care center
Source: PLoS One. 2026 Jan 27;21(1):e0341543. doi: 10.1371/journal.pone.0341543 (PMC12843566; doi:10.1371/journal.pone.0341543)
Supplement: S1 Table — (PDF) [file pone.0341543.s001.pdf]

Table 1. The American University of Beirut Medical Center intubation protocol

**A-Activation of intubation alert**
1-A multidisciplinary team reports to the scene.
2-Availability of videolaryngoscope, different airway aids, front-of-neck access equipment, anesthesia drugs, and vasopressors.
**B-Intubation protocol**
1-Preoxygenation during 5 min with O2 face mask
2-Premeptive use of vasopressors if the patient is hypotensive
3-Rapid sequence induction with systematic administration of muscle relaxants unless the provider decides otherwise
4-Preparation of post-intubation sedation and protective lung ventilation
5-Activation of difficult airway team and escalation to a more experienced provider after 2 failed intubation attempts
