# Supplementary material for: Adult airway management in ICU and regular floors: A prospective study as part of a healthcare improvement initiative in a tertiary care center
Source: PLoS One. 2026 Jan 27;21(1):e0341543. doi: 10.1371/journal.pone.0341543 (PMC12843566; doi:10.1371/journal.pone.0341543)
Supplement: S2 Table — (PDF) [file pone.0341543.s002.pdf]

Table 2: Code team members and difficult airway team members

**A- Code team members**
-Postgraduate year 3 on-call Medical/Surgical
-Senior anesthesia resident (Postgraduate year 4)
-Two registered nurses from the critical care units
-Respiratory Therapist
-Anesthesia nurse
-Any additional support personnel to be recruited by the team leader
**B- Difficult airway team members**
-Anesthesia faculty member (team lead)
-Senior anesthesia resident (Postgraduate year 4)
-Respiratory Therapist
-Anesthesia nurse
-Otorhinolaryngology senior resident (Postgraduate year 4)
